# Methyl 2-[2-(6-chloro­pyrimidin-4-yl­oxy)phen­yl]-3,3-dimethoxy­propanoate

**DOI:** 10.1107/S1600536809030736

**Published:** 2009-08-08

**Authors:** Chao Sheng, Qing-Bing Xu, Yuan-Yuan Liu, Hong-Jun Zhu

**Affiliations:** a Department of Applied Chemistry, College of Science, Nanjing University of Technology, Nanjing 210009, People’s Republic of China

## Abstract

In the title compound, C_16_H_17_ClN_2_O_5_, the dihedral angle between the aromatic rings is 77.36 (4)°. An intra­molecular C—H⋯O inter­action results in the formation of a planar [r.m.s. deviation = 0.103 (2) Å] five-membered ring, which is oriented at a dihedral angle of 4.84 (4)° with respect to the adjacent benzene ring. In the crystal structure, weak intermolecular C—H⋯π inter­actions are found.

## Related literature

For a related structure, see: Bowden & Brown (1996 ▶). For bond-length data, see: Allen *et al.* (1987 ▶).
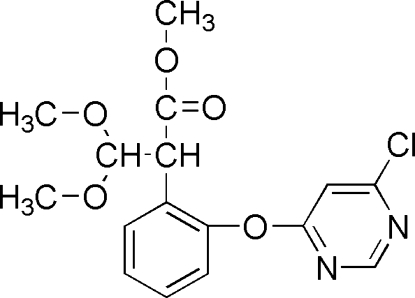


## Experimental

### 

#### Crystal data

C_16_H_17_ClN_2_O_5_
*M* *_r_* = 352.77
Triclinic, 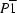

*a* = 9.5030 (19) Å
*b* = 10.051 (2) Å
*c* = 11.162 (2) Å
α = 101.24 (3)°
β = 108.47 (3)°
γ = 113.42 (3)°
*V* = 862.6 (5) Å^3^
*Z* = 2
Mo *K*α radiation
μ = 0.25 mm^−1^
*T* = 294 K
0.20 × 0.20 × 0.05 mm

#### Data collection

Enraf–Nonius CAD-4 diffractometer
Absorption correction: ψ scan (North *et al.*, 1968 ▶) *T*_min_ = 0.952, *T*_max_ = 0.988
3346 measured reflections
3140 independent reflections
1427 reflections with *I* > 2σ(*I*)
*R*_int_ = 0.035
3 standard reflections frequency: 120 min intensity decay: 1%

#### Refinement

*R*[*F* ^2^ > 2σ(*F* ^2^)] = 0.078
*wR*(*F* ^2^) = 0.173
*S* = 1.07
3140 reflections
211 parameters
H-atom parameters constrained
Δρ_max_ = 0.43 e Å^−3^
Δρ_min_ = −0.45 e Å^−3^

### 

Data collection: *CAD-4 Software* (Enraf–Nonius, 1985 ▶); cell refinement: *CAD-4 Software*; data reduction: *XCAD4* (Harms & Wocadlo, 1995 ▶); program(s) used to solve structure: *SHELXS97* (Sheldrick, 2008 ▶); program(s) used to refine structure: *SHELXL97* (Sheldrick, 2008 ▶); molecular graphics: *SHELXTL* (Sheldrick, 2008 ▶); software used to prepare material for publication: *SHELXTL* and *PLATON* (Spek, 2009 ▶).

## Supplementary Material

Crystal structure: contains datablocks I, global. DOI: 10.1107/S1600536809030736/hk2749sup1.cif

Structure factors: contains datablocks I. DOI: 10.1107/S1600536809030736/hk2749Isup2.hkl

Additional supplementary materials:  crystallographic information; 3D view; checkCIF report

## Figures and Tables

**Table 1:** Hydrogen-bond geometry (Å, °)

*D*—H⋯*A*	*D*—H	H⋯*A*	*D*⋯*A*	*D*—H⋯*A*
C6—H6*A*⋯O5	0.98	2.25	2.777 (6)	113
C1—H1*B*⋯*Cg*2^i^	0.96	2.97	3.696 (5)	134
C16—H16*A*⋯*Cg*1^i^	0.93	2.85	3.661 (4)	146

Symmetry code: (i) 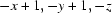. *Cg*1 and *Cg*2 are centroids of the C7–C12 and N1/N2/C13–C16 rings, respectively.

**Fig. 1.**
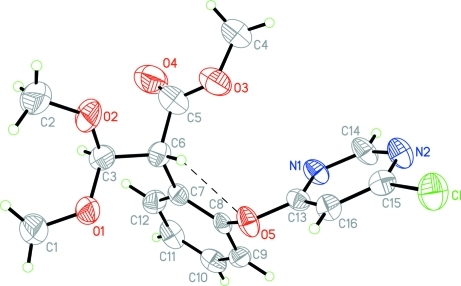
The molecular structure of the title molecule with the atom-numbering scheme. Displacement ellipsoids are drawn at the 50% probability level. Hydrogen bond is shown as dashed line.

## supplementary crystallographic information

### Comment

The title compound can be used as an intermediate in the preparation of azoxystrobin, which is an important fungicide (Bowden & Brown, 1996). We report herein the crystal structure of the title compound, which is of interest to us in the field.

In the molecule of the title compound (Fig. 1), the bond lengths (Allen *et al.*,  1987) and angles are within normal ranges. Rings A (C7-C12) and B (N1/N2/C13-C16) are, of course, planar and the dihedral angle between them is A/B = 77.36 (4)°. Intramolecular C-H···O interaction (Table 1) results in the formation of a planar five-membered ring C (O5/C6-C8/H6A), which is oriented with respect to the adjacent ring A at a dihedral angle of A/C = 4.84 (4)°.

In the crystal structure, weak C—H···π interactions (Table 1) are found.

### Experimental

The title compound was prepared acording to a literature method (Bowden & Brown, 1996). Crystals suitable for X-ray analysis were obtained by dissolving the title compound in methanol and evaporating the solvent slowly at room temperature for 8 d.

### Refinement

H atoms were positioned geometrically with C-H = 0.93, 0.98 and 0.96 Å for aromatic, methine and methyl H atoms, respectively, and constrained to ride on their parent atoms, with U_iso_(H) = xU_eq_(C), where x = 1.5 for methyl H and x = 1.2 for all other H atoms.

### Crystal data

**Table:**

C_16_H_17_ClN_2_O_5_	*Z* = 2
*M**_r_* = 352.77	*F*(000) = 368
Triclinic, *P*1	*D*_x_ = 1.358 Mg m^−^^3^
Hall symbol: -P 1	Mo *K*α radiation, λ = 0.71073 Å
*a* = 9.5030 (19) Å	Cell parameters from 25 reflections
*b* = 10.051 (2) Å	θ = 9–12°
*c* = 11.162 (2) Å	µ = 0.25 mm^−^^1^
α = 101.24 (3)°	*T* = 294 K
β = 108.47 (3)°	Needle, colorless
γ = 113.42 (3)°	0.20 × 0.20 × 0.05 mm
*V* = 862.6 (5) Å^3^	

### Data collection

**Table:**

Enraf–Nonius CAD-4 diffractometer	1427 reflections with *I* > 2σ(*I*)
Radiation source: fine-focus sealed tube	*R*_int_ = 0.035
graphite	θ_max_ = 25.3°, θ_min_ = 2.1°
ω/2θ scans	*h* = 0→11
Absorption correction: ψ scan (North *et al.*, 1968)	*k* = −12→11
*T*_min_ = 0.952, *T*_max_ = 0.988	*l* = −13→12
3346 measured reflections	3 standard reflections every 120 min
3140 independent reflections	intensity decay: 1%

### Refinement

**Table:**

Refinement on *F*^2^	Secondary atom site location: difference Fourier map
Least-squares matrix: full	Hydrogen site location: inferred from neighbouring sites
*R*[*F*^2^ > 2σ(*F*^2^)] = 0.078	H-atom parameters constrained
*wR*(*F*^2^) = 0.173	*w* = 1/[σ^2^(*F*_o_^2^) + (0.060*P*)^2^] where *P* = (*F*_o_^2^ + 2*F*_c_^2^)/3
*S* = 1.07	(Δ/σ)_max_ < 0.001
3140 reflections	Δρ_max_ = 0.43 e Å^−^^3^
211 parameters	Δρ_min_ = −0.45 e Å^−^^3^
Primary atom site location: structure-invariant direct methods	

### Special details

**Table:**

Geometry. All e.s.d.'s (except the e.s.d. in the dihedral angle between two l.s. planes) are estimated using the full covariance matrix. The cell e.s.d.'s are taken into account individually in the estimation of e.s.d.'s in distances, angles and torsion angles; correlations between e.s.d.'s in cell parameters are only used when they are defined by crystal symmetry. An approximate (isotropic) treatment of cell e.s.d.'s is used for estimating e.s.d.'s involving l.s. planes.
Refinement. Refinement of *F*^2^ against ALL reflections. The weighted *R*-factor *wR* and goodness of fit *S* are based on *F*^2^, conventional *R*-factors *R* are based on *F*, with *F* set to zero for negative *F*^2^. The threshold expression of *F*^2^ > σ(*F*^2^) is used only for calculating *R*-factors(gt) *etc*. and is not relevant to the choice of reflections for refinement. *R*-factors based on *F*^2^ are statistically about twice as large as those based on *F*, and *R*- factors based on ALL data will be even larger.

### Fractional atomic coordinates and isotropic or equivalent isotropic displacement parameters (Å^2^)

**Table:**

	*x*	*y*	*z*	*U*_iso_*/*U*_eq_	
Cl	0.16863 (16)	0.54848 (17)	0.23573 (15)	0.0918 (5)	
O1	−0.7131 (4)	0.4178 (4)	−0.3468 (3)	0.0806 (10)	
O2	−0.6406 (5)	0.2829 (5)	−0.4869 (4)	0.1119 (14)	
O3	−0.5192 (5)	0.0798 (5)	−0.3027 (4)	0.1150 (14)	
O4	−0.7857 (7)	−0.0410 (6)	−0.4408 (5)	0.1371 (18)	
O5	−0.4458 (3)	0.3794 (3)	−0.0203 (3)	0.0658 (9)	
N1	−0.3799 (5)	0.1982 (4)	0.0434 (4)	0.0631 (11)	
N2	−0.0880 (5)	0.2702 (5)	0.1614 (4)	0.0727 (11)	
C1	−0.8435 (9)	0.4438 (9)	−0.4217 (6)	0.131 (3)	
H1B	−0.8231	0.5446	−0.3730	0.196*	
H1C	−0.9517	0.3648	−0.4345	0.196*	
H1D	−0.8443	0.4398	−0.5086	0.196*	
C2	−0.6907 (10)	0.2967 (8)	−0.6060 (6)	0.140 (3)	
H2B	−0.6124	0.2972	−0.6432	0.210*	
H2C	−0.6938	0.3925	−0.5950	0.210*	
H2D	−0.8026	0.2105	−0.6664	0.210*	
C3	−0.7194 (7)	0.2788 (5)	−0.4025 (5)	0.0710 (14)	
H3A	−0.8400	0.2004	−0.4578	0.085*	
C4	−0.5126 (8)	−0.0639 (7)	−0.3384 (6)	0.1142 (16)	
H4A	−0.3983	−0.0432	−0.2896	0.171*	
H4B	−0.5469	−0.1044	−0.4343	0.171*	
H4C	−0.5883	−0.1389	−0.3148	0.171*	
C5	−0.6598 (11)	0.0736 (9)	−0.3573 (8)	0.1142 (16)	
C6	−0.6513 (6)	0.2253 (6)	−0.2981 (5)	0.0688 (13)	
H6A	−0.5298	0.3025	−0.2460	0.083*	
C7	−0.7250 (6)	0.2132 (5)	−0.1948 (5)	0.0517 (11)	
C8	−0.6183 (5)	0.2823 (4)	−0.0593 (5)	0.0464 (10)	
C9	−0.6753 (6)	0.2753 (5)	0.0390 (5)	0.0609 (13)	
H9A	−0.5986	0.3259	0.1305	0.073*	
C10	−0.8481 (7)	0.1921 (6)	0.0000 (6)	0.0699 (14)	
H10A	−0.8884	0.1850	0.0656	0.084*	
C11	−0.9602 (6)	0.1202 (6)	−0.1340 (7)	0.0793 (16)	
H11A	−1.0770	0.0644	−0.1607	0.095*	
C12	−0.8968 (6)	0.1316 (5)	−0.2302 (5)	0.0691 (14)	
H12A	−0.9732	0.0823	−0.3218	0.083*	
C13	−0.3283 (6)	0.3428 (5)	0.0417 (4)	0.0536 (11)	
C14	−0.2533 (6)	0.1731 (5)	0.1042 (5)	0.0720 (14)	
H14A	−0.2858	0.0737	0.1068	0.086*	
C15	−0.0453 (6)	0.4133 (5)	0.1578 (4)	0.0586 (12)	
C16	−0.1620 (5)	0.4550 (5)	0.0963 (4)	0.0575 (12)	
H16A	−0.1295	0.5538	0.0923	0.069*	

### Atomic displacement parameters (Å^2^)

**Table:**

	*U*^11^	*U*^22^	*U*^33^	*U*^12^	*U*^13^	*U*^23^
Cl	0.0588 (8)	0.0939 (11)	0.1023 (11)	0.0264 (7)	0.0267 (8)	0.0357 (8)
O1	0.104 (3)	0.067 (2)	0.066 (2)	0.047 (2)	0.023 (2)	0.0268 (18)
O2	0.161 (4)	0.177 (4)	0.075 (3)	0.115 (3)	0.076 (3)	0.085 (3)
O3	0.118 (3)	0.119 (3)	0.127 (3)	0.084 (3)	0.050 (3)	0.033 (3)
O4	0.137 (4)	0.122 (3)	0.136 (4)	0.083 (3)	0.037 (3)	0.013 (3)
O5	0.0501 (19)	0.0528 (18)	0.097 (2)	0.0245 (16)	0.0248 (17)	0.0461 (18)
N1	0.065 (2)	0.042 (2)	0.092 (3)	0.0293 (19)	0.037 (2)	0.035 (2)
N2	0.064 (3)	0.073 (3)	0.099 (3)	0.039 (2)	0.039 (2)	0.050 (3)
C1	0.169 (7)	0.195 (7)	0.096 (5)	0.143 (6)	0.064 (5)	0.062 (5)
C2	0.239 (9)	0.150 (6)	0.093 (5)	0.133 (6)	0.082 (6)	0.067 (5)
C3	0.100 (4)	0.053 (3)	0.064 (3)	0.036 (3)	0.039 (3)	0.028 (3)
C4	0.127 (4)	0.126 (4)	0.125 (4)	0.092 (3)	0.056 (3)	0.047 (3)
C5	0.127 (4)	0.126 (4)	0.125 (4)	0.092 (3)	0.056 (3)	0.047 (3)
C6	0.084 (3)	0.078 (3)	0.058 (3)	0.046 (3)	0.034 (3)	0.032 (2)
C7	0.059 (3)	0.044 (2)	0.060 (3)	0.029 (2)	0.027 (3)	0.027 (2)
C8	0.051 (3)	0.038 (2)	0.057 (3)	0.027 (2)	0.020 (3)	0.025 (2)
C9	0.090 (4)	0.045 (3)	0.053 (3)	0.037 (3)	0.030 (3)	0.024 (2)
C10	0.085 (4)	0.061 (3)	0.104 (5)	0.044 (3)	0.063 (4)	0.053 (3)
C11	0.048 (3)	0.063 (3)	0.120 (5)	0.021 (3)	0.032 (4)	0.044 (4)
C12	0.068 (4)	0.054 (3)	0.065 (3)	0.023 (3)	0.019 (3)	0.014 (3)
C13	0.062 (3)	0.048 (3)	0.062 (3)	0.032 (2)	0.031 (2)	0.025 (2)
C14	0.068 (3)	0.053 (3)	0.113 (4)	0.039 (3)	0.041 (3)	0.044 (3)
C15	0.062 (3)	0.059 (3)	0.060 (3)	0.025 (3)	0.036 (3)	0.027 (2)
C16	0.053 (3)	0.048 (3)	0.068 (3)	0.021 (2)	0.027 (3)	0.026 (2)

### Geometric parameters (Å, °)

**Table:**

Cl—C15	1.720 (5)	C3—H3A	0.9800
O1—C1	1.410 (6)	C4—H4A	0.9600
O1—C3	1.383 (5)	C4—H4B	0.9600
O2—C2	1.318 (6)	C4—H4C	0.9600
O2—C3	1.373 (5)	C5—C6	1.498 (8)
O3—C4	1.453 (6)	C6—C7	1.528 (6)
O3—C5	1.250 (7)	C6—H6A	0.9800
O4—C5	1.186 (8)	C7—C8	1.363 (5)
O5—C8	1.393 (4)	C7—C12	1.376 (6)
O5—C13	1.341 (5)	C8—C9	1.370 (6)
N1—C13	1.343 (5)	C9—C10	1.378 (6)
N1—C14	1.327 (5)	C9—H9A	0.9300
N2—C14	1.316 (5)	C10—C11	1.361 (7)
N2—C15	1.343 (5)	C10—H10A	0.9300
C1—H1B	0.9600	C11—C12	1.390 (7)
C1—H1C	0.9600	C11—H11A	0.9300
C1—H1D	0.9600	C12—H12A	0.9300
C2—H2B	0.9600	C13—C16	1.359 (5)
C2—H2C	0.9600	C14—H14A	0.9300
C2—H2D	0.9600	C15—C16	1.370 (5)
C3—C6	1.452 (6)	C16—H16A	0.9300
			
C3—O1—C1	117.8 (4)	C5—C6—C7	108.8 (4)
C2—O2—C3	126.5 (5)	C3—C6—H6A	106.1
C5—O3—C4	117.8 (5)	C5—C6—H6A	106.1
C13—O5—C8	121.1 (3)	C7—C6—H6A	106.1
C14—N1—C13	114.1 (4)	C8—C7—C12	116.5 (4)
C14—N2—C15	114.3 (4)	C8—C7—C6	119.9 (4)
O1—C1—H1B	109.5	C12—C7—C6	123.6 (4)
O1—C1—H1C	109.5	C7—C8—C9	123.0 (4)
H1B—C1—H1C	109.5	C7—C8—O5	117.6 (4)
O1—C1—H1D	109.5	C9—C8—O5	118.9 (4)
H1B—C1—H1D	109.5	C8—C9—C10	118.9 (4)
H1C—C1—H1D	109.5	C8—C9—H9A	120.5
O2—C2—H2B	109.5	C10—C9—H9A	120.5
O2—C2—H2C	109.5	C11—C10—C9	120.4 (5)
H2B—C2—H2C	109.5	C11—C10—H10A	119.8
O2—C2—H2D	109.5	C9—C10—H10A	119.8
H2B—C2—H2D	109.5	C10—C11—C12	118.7 (5)
H2C—C2—H2D	109.5	C10—C11—H11A	120.6
O2—C3—O1	114.2 (4)	C12—C11—H11A	120.6
O2—C3—C6	109.9 (4)	C7—C12—C11	122.4 (5)
O1—C3—C6	111.5 (4)	C7—C12—H12A	118.8
O2—C3—H3A	107.0	C11—C12—H12A	118.8
O1—C3—H3A	107.0	O5—C13—N1	119.0 (4)
C6—C3—H3A	107.0	O5—C13—C16	117.2 (4)
O3—C4—H4A	109.5	N1—C13—C16	123.9 (4)
O3—C4—H4B	109.5	N2—C14—N1	128.5 (4)
H4A—C4—H4B	109.5	N2—C14—H14A	115.7
O3—C4—H4C	109.5	N1—C14—H14A	115.7
H4A—C4—H4C	109.5	N2—C15—C16	123.5 (4)
H4B—C4—H4C	109.5	N2—C15—Cl	116.9 (4)
O4—C5—O3	123.8 (7)	C16—C15—Cl	119.6 (4)
O4—C5—C6	124.5 (7)	C13—C16—C15	115.6 (4)
O3—C5—C6	111.6 (7)	C13—C16—H16A	122.2
C3—C6—C5	112.0 (5)	C15—C16—H16A	122.2
C3—C6—C7	117.1 (4)		
			
C2—O2—C3—O1	−67.6 (7)	C13—O5—C8—C7	−114.9 (4)
C2—O2—C3—C6	166.3 (6)	C13—O5—C8—C9	72.8 (5)
C1—O1—C3—O2	87.6 (6)	C7—C8—C9—C10	1.4 (6)
C1—O1—C3—C6	−147.2 (5)	O5—C8—C9—C10	173.3 (3)
C4—O3—C5—O4	−3.6 (11)	C8—C9—C10—C11	−1.0 (6)
C4—O3—C5—C6	173.0 (5)	C9—C10—C11—C12	0.4 (7)
O2—C3—C6—C5	−53.2 (6)	C8—C7—C12—C11	0.5 (6)
O1—C3—C6—C5	179.1 (5)	C6—C7—C12—C11	178.8 (4)
O2—C3—C6—C7	−179.9 (4)	C10—C11—C12—C7	−0.1 (7)
O1—C3—C6—C7	52.5 (6)	C8—O5—C13—N1	12.4 (6)
O4—C5—C6—C3	−54.2 (10)	C8—O5—C13—C16	−169.3 (4)
O3—C5—C6—C3	129.3 (6)	C14—N1—C13—O5	178.4 (4)
O4—C5—C6—C7	76.8 (8)	C14—N1—C13—C16	0.2 (6)
O3—C5—C6—C7	−99.7 (6)	C15—N2—C14—N1	0.9 (8)
C3—C6—C7—C8	−128.5 (4)	C13—N1—C14—N2	0.0 (8)
C5—C6—C7—C8	103.3 (5)	C14—N2—C15—C16	−2.0 (7)
C3—C6—C7—C12	53.2 (6)	C14—N2—C15—Cl	177.9 (4)
C5—C6—C7—C12	−75.0 (6)	O5—C13—C16—C15	−179.4 (4)
C12—C7—C8—C9	−1.1 (6)	N1—C13—C16—C15	−1.2 (7)
C6—C7—C8—C9	−179.5 (4)	N2—C15—C16—C13	2.2 (7)
C12—C7—C8—O5	−173.1 (3)	Cl—C15—C16—C13	−177.7 (3)
C6—C7—C8—O5	8.4 (5)		

### Hydrogen-bond geometry (Å, °)

**Table:**

*D*—H···*A*	*D*—H	H···*A*	*D*···*A*	*D*—H···*A*
C6—H6A···O5	0.98	2.25	2.777 (6)	113
C1—H1B···Cg2^i^	0.96	2.97	3.696 (5)	134
C16—H16A···Cg1^i^	0.93	2.85	3.661 (4)	146

Symmetry codes: (i) −*x*+1, −*y*+1, −*z*.
